# Preference of harmonic loudness degree on piano timbre

**DOI:** 10.3389/fpsyg.2022.908543

**Published:** 2022-09-29

**Authors:** Yuxiang Cai, Yushi Ling, Guikang Cao, Xuefeng Zhou

**Affiliations:** ^1^Music College, Southwest University, Chongqing, China; ^2^Faculty of Psychology, Southwest University, Chongqing, China

**Keywords:** piano timbre, equalizer, harmonics loudness, audio preference, digital music

## Abstract

This study exploratively conducted two investigations of timbre influenced by harmonic loudness. Investigation 1 examined piano timbre preference between non-processed melodies and two kinds of adjustments created *via* an equalizer–a basic tool used for sound engineering and for producing audio materials. Using the paired comparison method, 98 respondents were surveyed. The results show that in the bass range of the piano sound, the preferred audio material was that produced *via* an equalizer by reducing the loudness of the 7th and 9th harmonics by 15 dB, while enhancing the loudness of the 8th harmonic by 15 dB. Investigation 2 examined three degrees of the processed melodies—adjusting the harmonics by 15, 10, and 5 dB. The results show that the 15 dB change was the best, while the 10 dB change was the worst. These results provide a new approach to improving the sound timbre for sound engineering and artificial intelligence music production.

## Introduction

Timbre is not a consciously processed dimension of the image, although some composers and orchestrators’ knowledge is declarative “(for instance, knowing in the abstract that particular sounds blend well), the remainder is presumably based on inner hearing or ‘musical imagery’.” “A pilot experiment manipulated instrumentation, while the main experiment manipulated sound filters. The hypothesis that participants are able to internalize timbral aspects of music was supported by an ability to perform the timbre discrimination task, and by facilitated response when imaging the timbre context compared with non-imaging.” The results suggest that timbre may be a sound characteristic that is optionally present in imagery for music (Bailes, 2007, p. 21). Up to now, the view of timbre as subjective music imagery is still supported by some musicians. After all, timbre perception is still a complex issue in both theory and practice. In the field of theory, timbre is thought of as “a misleadingly simple and exceedingly vague word encompassing a very complex set of auditory attributes, as well as a plethora of intricate psychological and musical issues” (McAdams and Giordano, 2008, p. 72). Those intricate issues relate to many parameters of perception that are not accounted for by pitch, loudness, spatial position, or duration (McAdams, 1999, p. 85).

From a psychoacoustics view, the parameters—attack time, spectral centroid, and spectrum fine structure—appear as major determinants of timbre (Caclin et al., 2005, p. 471). Timbre is a multidimensional perceptual attribute with multiple underlying acoustic dimensions of both temporal and spectral types (Caclin et al., 2007, p. 159). In respect to spectral type, using event-related potentials (ERPs), Caclin et al. examined the processing of auditory dimensions in sensory memory and found that interactive behavior occurred in the pair with two spectral dimensions—spectral center of gravity (SCG) and even harmonic attenuation (EHA) (Caclin et al., 2006, p. 1968–1969). Early ERP effects (before 200 ms) of the condition factor were observed for the SCG/EHA, and the effect of the condition factor is more pronounced after 500 ms; before 200 ms, there was a significant Condition × Congruency interaction, “in the 250–350 ms latency range, a significant congruency effect is observed, and slightly later (280–400 ms), a significant Condition × Congruency interaction arises for this pair of dimensions” (Caclin et al., 2008, p. 55–58). In respect to materials, using a categorization task, wood, metal and glass were examined; the results revealed that the processing of metal sounds differed significantly from glass and wood sounds as early as 150 ms and up to 700 ms (Aramaki et al., 2009, p. 1). This research advanced the understanding of perceptions of timbre.

Besides understanding perceptions of timbre, when focusing on piano manufacturing technology, many researchers have tried to better understand experimental results of hammer-string interaction, and to obtain a better agreement between the experimental and theoretical results. The main points of interest in the hammer-string interaction are: (1) time pattern of the force acting between the hammer and the string; (2) contact time; (3) temporary loss of contact between the hammer and the string; (4) spectral pattern of partials; and (5) energy transfer efficiency (Suzuki and Nakamura, 1990, p. 158).

The spectral pattern of partials can be taken as a spectrum fine structure “related to the local shape of the spectrum, modeled by even-harmonic attenuation” (Caclin et al., 2005, p. 481). Turning to the piano manufacturing theory corresponding to spectral pattern of partials, one approach—using hammer positions at a cord length of 1/8—controls the 7th and 9th discord harmonics while decreasing the loudness of the 8th concord harmonic (Li, 1992, p. 14–17). The relationship between the root and the 7th, 8th, and 9th harmonics can be explained by the following two examples. C root’s 7th, 8th, and 9th harmonics correspond to B-flat, C, and D, while D root’s 7th, 8th, and 9th harmonics correspond to C, D, and E. Many piano manufacturers have used Young’s Law to select hammer positions at string lengths of 1/7, 1/8, 1/9, and so on, in the mid-to-low-range pitch of the piano to improve piano timbre by controlling the inconsistent harmonic loudness (Jin et al., 2002, p. 5–6). However, contemporary recording techniques (equalizers) used for timbre adjustment can be employed without sacrificing harmonic loudness (Ramo and Valimaki, 2014, p. 302–303). Thus, the following question arises: can ordinary listeners, once used to hearing natural piano timbre, perceive changes in timbre processed *via* an equalizer?

A study conducted by Huang and Xie (2005, p. 4–5) used a real piano (the Zhujiang brand made in China) and equalizers processing the 7th and 9th harmonics of each sound. When the 7th and 9th harmonic loudness was attenuated by 6 dB, its sound quality was highly praised by the tuner respondents. If the 7th and 9th harmonic loudness was attenuated by 10 dB–20 dB, the tuner respondents thought that the sound became thin. However, the survey used in this study suffered from several limitations: (1) the sample size was small, with only six and seven subjects recruited in the first and follow-up surveys, respectively; (2) the survey only used isolated sounds rather than music clips as audio materials, which is different from the real experience of subjects listening to music; and (3) the investigation did not limit the sound range; that is, in the process of the piano making the chord, the point design of each range was not the same, so failing to limit the range and treating all sounds the same will bias the results. The present study addresses these limitations through four measures: (1) including 146 respondents; (2) employing a phrase written specifically for this survey instead of using isolated sounds; (3) selecting audio materials from the sound area where the striking point is 1/8 of the string length (piano production loses the 8th harmonic intensity in this sound area, which can be improved using equalizer technology); and (4) the sound was not directly obtained by piano but created using Spectrasonics’s KeyScape 1.1 piano sound source software (Steinberg Cubase Art 10 Digital Audio Workstation), wherein we employed a customized piano Yamaha C7 to provide the character control function. The KeyScape piano sound source software is very popular among composers and recording engineers; it is widely used in music production fields, such as film and game music.

In this case, combining the theory of parameters of perception and spectrum structure, in our study of timbre, we defined loudness as the intensity shown by the equalizer.

This study explored the influence of harmonic loudness—manipulated by equalizer technology—on piano timbre preference. The research consisted of two investigations: (1) harmonics with the non-processed and processed sounds; and (2) three degrees of the processed harmonics.

## Investigation 1 (harmonics with the non-processed and processed sounds)

### Investigation 1: Materials and methods

We conducted a survey of 98 people (females = 75; mean age = 20.7 years; musicians = 30). Convenience sampling was employed, and subjects were recruited from the student population at Southwest University. Due to the difference in the number of male vs. female respondents, the survey results were also analyzed by gender. The musician respondents were majoring in bassoon, cello, clarinet, double bass, electric guitar, erhu violin, piano, pipa, vocal music, or zither. The average number of years they had spent learning music was 8.8.

**FIGURE 1:**
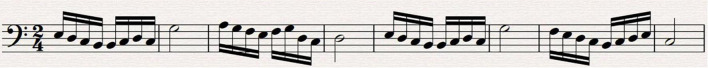
20-Second piano melody written for this study. © 2022 IEEE. Reprinted with permission from Cai and Zhou (2021).

The audio melody (Figure 1) used in the study was composed in the bass range (from 123.47 to 220 Hz)—equivalent to the hammer position at 1/8 of the string length—of the piano sound source software. An equalizer was used to adjust the harmonic intensity of a specific number of individual pitches in the audio material. The 20-s length melody comprised two phrases which were in the same key of C major. The first phrase ended on the dominant and the second phrase ended on the tonic. The melodic intervals included steps and jumps, while the rhythmic patterns included sixteenth notes (short tones) and half notes (long tones). Since piano timbre is based on two processes—percussion and continuation—this investigation manipulated continuation with short and long tones, which made it possible to adjust it to different schemes.

**FIGURE 2:**
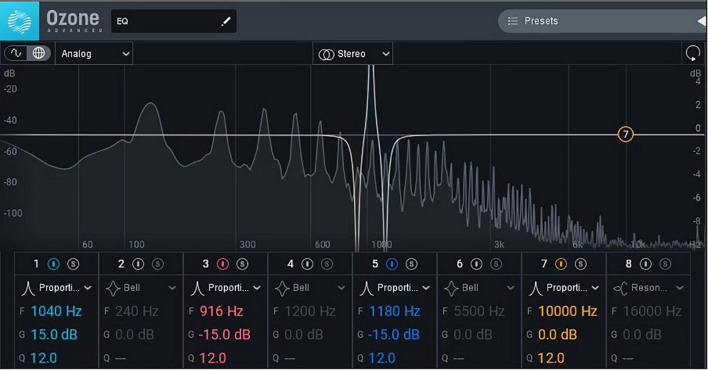
Effect drawing of harmonic loudness adjustment. Image reproduced with permission from Ozone software. © 2022 IEEE. Reprinted with permission from Cai and Zhou (2021).

The method applied when using the equalizer to adjust the 7th–9th harmonic loudness of each tone in the audio materials was as follows. Using Steinberg Cubase Art 10, after each tone had been independently divided into a sound track, the equalizer software (iZotope Ozone 9.1) was opened, the adjustment mode was set to Proportional Q, and the *Q* value was set to the maximum value of 12. The larger the *Q* value, the smaller the adjusted frequency range and the more accurate the adjustment. The loudness frequency was then repeatedly adjusted on the F parameter. After determining the frequency with the best effect, the harmonic loudness could be accurately adjusted (Figure 2).

**FIGURE 3:**
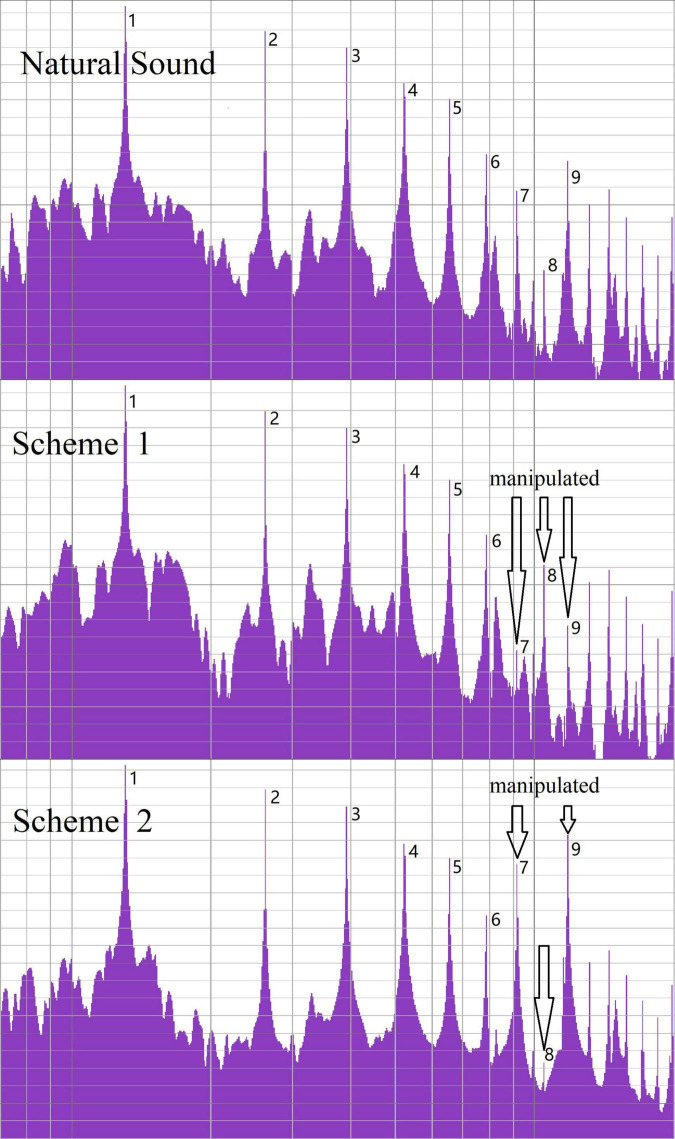
Harmonic loudness in investigation 1.

According to the paired comparison method (Tarrow, 2010, p. 232–233), each of the five factors—audio materials A, B, C, D, and E—was paired with each of the other audio materials side-by-side. Factor A enhanced the 8th harmonic by 15 dB—the decibel value was used to describe the sound level—while the 7th and 9th harmonics were reduced by 15 dB (Huang and Xie, 2005, p. 5). Factor B comprised the non-processed sound—the original sound output under the piano sound source software default settings. Factor C enhanced the 8th harmonic by 15 dB, while the 7th and 9th harmonics were reduced by 15 dB on short tones; in addition, the 7th and 9th harmonics were enhanced by 15 dB while reducing the 8th harmonic by 15 dB on long tones (Figure 3). Factor D enhanced the 7th and 9th harmonics by 15 dB while reducing the 8th harmonic by 15 dB on short tones; simultaneously, the 8th harmonic was enhanced by 15 dB, while the 7th and 9th harmonics were reduced by 15 dB on long tones. Finally, Factor E enhanced the 7th and 9th harmonics by 15 dB while reducing the 8th harmonic by 15 dB on both short and long tones (Table 1). All ten pairs (AB, AC, AD, AE, BC, BD, BE, CD, CE, and DE) were obtained.

**TABLE 1:** Factors used for each of the five audio materials in investigation 1.

Factors	Schemes
A	Enhancing the 8th harmonic by 15 dB while the 7th and 9th harmonics were reduced by 15 dB
B	No processing
C	Enhancing the 8th harmonic by 15 dB while the 7th and 9th harmonics were reduced by 15 dB in short tones; enhancing the 7th and 9th harmonics by 15 dB while the 8th harmonic was reduced by 15 dB in long tones
D	Enhancing the 7th and 9th harmonics by 15 dB while the 8th harmonic was reduced by 15 dB in short tones, enhancing the 8th harmonic by 15 dB while the 7th and 9th harmonics were reduced by 15 dB in long tones
E	Enhancing the 7th and 9th harmonics by 15 dB while the 8th harmonic was reduced by 15 dB

During the survey, random pairs of the whole melody with different schemes (Table 1) were played by a Hewlett-Packard 17-w119TX laptop. The respondents wore professional-grade Sony MDR-7,506 monitor headphones to listen to the audio and listened to each pair twice to test whether the participants chose a stable option. Respondents were then required to write down their preference.

Prior to the experiment, participants were told to listen to three sets of test audio, which ensured that they fully understood the investigation procedure. The formal survey took 30 min to complete. To minimize respondent fatigue, 5 min’ rest time was provided in the middle of the listening procedure.

### Investigation 1: Results

In line with previous studies, the difference analysis based on paired comparisons was performed using a Chi-square test (Saito, 1996, p. 36–37; Ge et al., 2005, p. 850). After the survey, we calculated the average number of times that respondents in each group (musician vs. non-musician) chose each audio used in the survey (Table 2). The Chi-square test revealed that the preferences of professional and non-professional subjects for audio materials did not differ significantly (*p* = 1.000).

**TABLE 2:** Frequency statistics of audio material preference analysis of musician and non-musician subjects.

Average selected frequency	A	B	C	D	E
Musician	3.63	3.13	1.70	0.97	0.57
Non-musician	3.16	2.71	1.75	1.46	0.93

Further Chi-square tests were conducted to determine differences in the preferences of musician and non-musician subjects for each audio material. The results indicated that only material A was close to statistical significance (*X*^2^ = 6.317, p = 0.097); there were no significant differences among the other materials (Table 3). At the same time, the Chi-square test was used to calculate the differences between male and female participants in the average selection frequency of five audio materials. The results (*p* = 1.000) suggested that the participants’ gender had no significant effect on their preference for audio materials.

**TABLE 3:** Chi-square test of preference of musician and non-musician subjects for audio material selection.

	A	B	C	D	E
*X* ^2^	6.317	6.667	5.240	5.485	5.390
*p*	0.097	0.155	0.264	0.241	0.250

**FIGURE 4:**
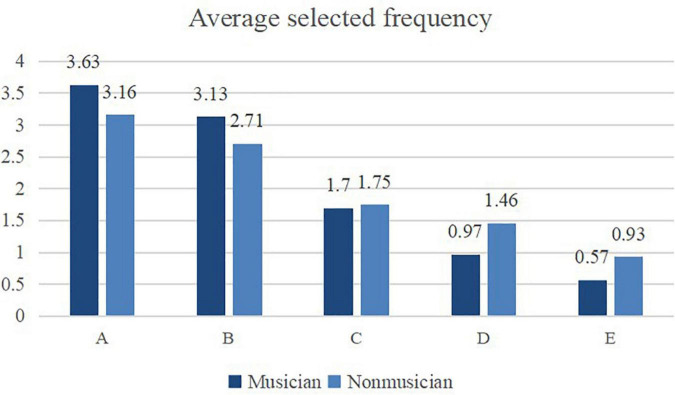
Frequency statistics of audio material preference analysis of musician and nonmusician subjects.

To investigate the differences in subjects’ preferences for the five kinds of audio, we conducted Chi-square tests on the selection frequency of musician vs. non-musician subjects and found that there were significant differences in musicians’ preferences for the five kinds of materials (*X*^2^ = 88.792, *p* < 0.001). There were also significant differences in the preferences of non-musicians for the five materials (*X*^2^ = 103.214, *p* < 0.001). This indicates that both musicians and non-musicians can distinguish the differences between the five audio materials. Specifically, musicians and non-musicians chose the A audio most frequently (*M* = 3.398, *SD* = 0.104), followed by the B audio (*M* = 2.920, *SD* = 0.104), C audio (*M* = 1.725, *SD* = 0.104), D audio (*M* = 1.211, *SD* = 0.104), and E audio (*M* = 0.747, *SD* = 0.104). The results also showed that the average selected frequencies of A, B, C, D, and E decreased in turn. Audio A received the highest number of votes, followed by audio B; likewise, audios C, D, and E received significantly fewer votes than the first two categories (Figure 4).

Regarding the short tones in the audio materials, we take audio materials A and C as a reference and compare them against the votes for audio materials D and E. The findings indicate that, compared with long tones, the short tones in audio A and C were preferred. This may suggest that listeners are more sensitive to sound quality when listening to continuous dense sound. Finally, Audio E was the least preferred by respondents, as expected.

Investigation 2 examines the specific value of overtone loudness and the critical point of tolerance.

## Investigation 2 (three degrees of the processed harmonics)

### Investigation 2: Materials and methods

In Investigation 2, the 20-s piano melody written specifically for this study (Figure 1) was again used as the manipulated base. We examined three degrees—adjusting harmonics by 15, 10, and 5 dB—of the processed harmonic and conducted another survey. Participants were again recruited from the student population at Southwest University; they included 48 musicians (females = 32; mean age = 20.5 years), of whom 28 were majoring in piano and 20 were majoring in marimba, saxophone, clarinet, guzheng, violin, or vocal music. The average number of years spent learning music was 9.5.

**FIGURE 5:**
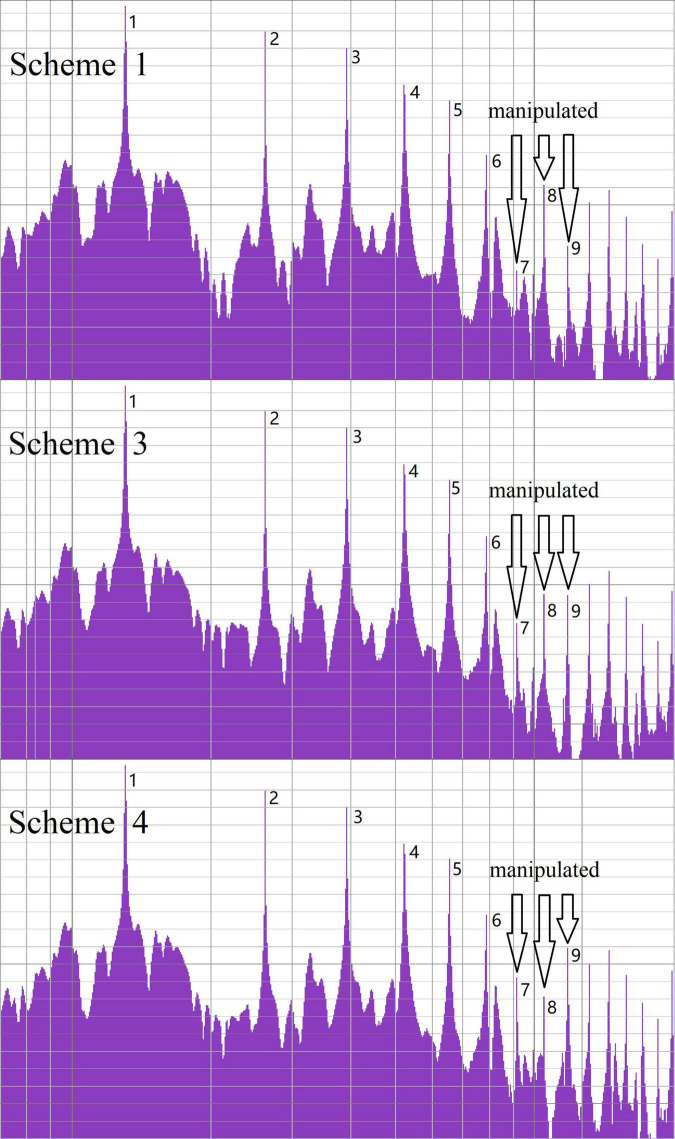
Harmonic loudness in investigation 2.

Each of the three audio materials (A, F, and G) was paired with other audio materials side-by-side. Factor A was the same as in Investigation 1. Factor F enhanced the 8th harmonic by 10 dB, while the 7th and 9th harmonics were reduced by 10 dB. Factor G enhanced the 8th harmonic by 5 dB, while the 7th and 9th harmonics were reduced by 5 dB (Figure 5). A total of six pairs (AF, FA, AG, GA, FG, and GF) were obtained.

During the survey, random pairs of the whole melody with different schemes (Table 4) were played by a Hewlett-Packard 17-w119TX laptop. The respondents wore professional-grade Sony MDR-7,506 monitor headphones to listen to the audio (again listening to each pair twice to test whether participants chose a stable option). Then they were required to write down their preference.

**TABLE 4:** Factors used for each of the three audio materials in investigation 2.

Factors	Schemes
A	Enhancing the 8th harmonic by 15 dB while the 7th and 9th harmonics were reduced by 15 dB
F	Enhancing the 8th harmonic by 10 dB while the 7th and 9th harmonics were reduced by 10 dB
G	Enhancing the 8th harmonic by 5 dB while the 7th and 9th harmonics were reduced by 5 dB

Prior to the experiment, participants listened to three sets of test audio, which ensured that they fully understood the investigation procedure. The formal survey took 15 min to complete. After the listening survey, the respondents were required to answer the following questions: (1) When listening, do you feel any differences between the two audio materials in each group? and (2) If there is any difference, can you describe the specific audio?

### Investigation 2: Results

As in the first survey, we calculated the average number of times each audio material was selected by the musician respondents (Table 5). Chi-square tests were performed for the selected frequencies of each audio, and the effect was significant (*X*^2^ = 40.042, *p* < 0.001). A Chi-square test also showed that there were again no significant gender differences (*p* = 1.000), which means that gender has no effect on subjects’ preferences for the audio materials.

**TABLE 5:** Average frequency of three audio materials selected in paired comparison.

	A	G	F
Average selected frequency	2.96	2.08	0.96

**FIGURE 6:**
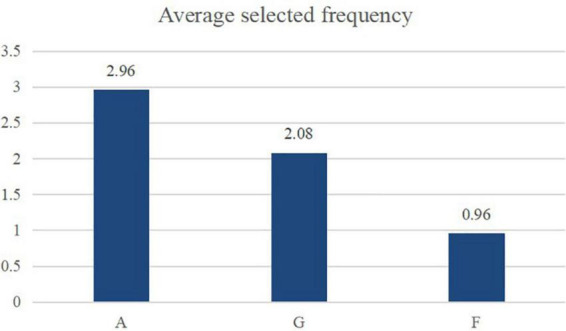
Average frequency of three audio materials selected in paired comparison.

The average selected frequency of each audio material shows that, among the three audio materials, audio A was still the most popular, followed by G and F, the latter of which received far fewer votes (Figure 6). Therefore, the procedure that enhanced the 8th harmonic by 15 dB and weakened the 7th and 9th harmonics by 15 dB was preferred by respondents. This finding supports the results of Investigation 1.

Here, Audio A (enhancing the 8th harmonic by 15 dB and weakening the 7th and 9th harmonics by 15 dB) was preferred by the respondents. This conclusion may benefit from further study using the isolated sounds view (Huang and Xie, 2005, p. 4–7) and phrase view.

Audio F (enhancing the 8th harmonic by 10 dB, while the 7th and 9th harmonics were reduced by 10 dB) received the lowest average selected frequency—only 0.96. Is this the critical point? The results of the interviews conducted after the listening survey might be close to this question.

Audio F’s corresponding descriptors were mostly “thin,” “rough,” and so on. Compared with audio A’s highly descriptive words—“delicate,” “clear,” and “ethereal”—and audio G’s descriptive words (e.g., “plump”), audio F was described as negative words and received a much lower number of votes. Compared with the results of the study by Huang and Xie (2005, p. 4–7)—which found that timbre was highly praised when the 7th and 9th harmonic loudness was attenuated by 6 dB and described as “thin” when the 7th and 9th harmonic loudness was attenuated by 10–20 dB—the aesthetic perceptions noted here are similar.

## General discussion

The two investigations conducted in this study did not directly reference other research in the fields of timbre theory, psychoacoustics, and timbre perception understanding. However, the designs of the investigations were based on the idea that timbre is not a simple issue accounted for by ordinary parameters; rather, its auditory effect might be improved. In this case, shaping harmonic loudness of the spectrum fine structure, examining example of patterns in “real” music (Vuust et al., 2012, p. 141), and exploring the sound characteristics, became three discussions as follows:

Firstly, shaping harmonic loudness. Comparing with the EHA model, the current investigations were designed to change the value of the 7th, 8th, and 9th harmonic loudness, the difference of which might be discussed. The current study attenuated and/or enhanced the value by 15, 10 or 5 dB, which resulted in a large-scale change for ascending and descending. Such a change was based on the study of Huang and Xie (2005, p. 4–5) who attenuated the 7th and 9th harmonics of a single sound by 6 dB or 10–20 dB. This way aims for practical application. While Caclin et al. (2006) did the EHA from 0 to 8 dB (2005, p. 480), 0 or–6.4 dB (2006, 1963), 0 or 10 dB (2008, p. 51), Tużnik et al. (2018) attenuated the EHA 5 or 10 dB (2018, p. 11). The EHA model contributed to the principle of timbre. Either in principle or application, does 10 dB serve as a threshold of negative timbre perception? This deserves further investigation.

Secondly, examining preference of phrase. Previous research—using audio samples produced by four piano performers on a real piano in a concert hall, which did not change the spectrum’s shape—found that (1) timbre preferences for single sounds might relate to different spectrum shapes of the 1st, 2nd, 3rd, 4th, and 5th harmonic intensities, as shown by the software Audacity; (2) no relation between the single sound and phrase was found (Zhou et al., 2018, p. 33–34). (1) Suggested that the perceived timbre might be a measurable item but not an abstract rule. (2) Suggested, as Clarke explained, “perception must be understood as a relationship between environmentally available information and the capacities, sensitivities, and interests of a perceiver” (Clarke, 2005, p. 91). The current study employs two phrases composed as real music, shaping the harmonic loudness by Spectrasonics’s KeyScape 1.1 piano sound source software and the equalizer. The investigations focused on the whole phrase and ignored the single sound, being inclined to a top-down processing method. Just as the study of musical syntactic perception pursues some abstract rule, this study, which took a phrase as the research object, also explored from an overall perspective. Although our exploration has not found abstract rules from the different design schemes of long and short tone conditions (please see Factors C and D in Table 1), it would be interesting to combine top-down attention and selective attention to enable effective encoding and maintenance of relevant information in memory (Lim et al., 2015, p. 16102).

Thirdly, exploring the sound characteristics. The results of interviews after investigation 2 can be seen as a description of phrase timbre characteristics. Although they cannot be quantified, some descriptors can reflect and be abstracted visual graphics. In some experiments, graphics presenting three different geometrical shapes (triangle, square and circle) each constituted a cue for the subject to induce auditory imagery of a specific sound (Tużnik et al., 2018, p. 11). However, the current study is not as good as the previous ERP study—highlighted in the introduction—in terms of detecting the time course of accurately perceiving sound characteristics. Besides, using the stimulus of Alberti Bass and rising and falling sequences of thirds, research found that jazz musicians overall have larger MMNs to the six sound deviants—pitch, timbre, location, intensity, slide and rhythm—than all other groups (the other three are band musicians, classical musicians and non-musicians). Jazz musicians learn and perform music to a great extent by ear, “furthermore, jazz music is characterized by complex chord changes, rich harmonies, and challenging rhythmic material, which may boost these performers’ theoretical and ear training skill”(Vuust et al., 2012, p. 145). That is to say, more sound characteristics need be studied; training benefits reflect distinct sensorimotor processing stages (Garner et al., 2015, p. 2091).

## Conclusion

This study conducted two investigations comprised of a melody phrase listening examination that manipulated timbre variables in the bass range of the piano sound. The following conclusions can be drawn. First, listeners’ preferences regarding piano timbre were more positive when equalizers were used to enhance the 8th harmonic by 15 dB while reducing the 7th and 9th harmonics by 15 dB. Second, participants least preferred the processed harmonics when we enhanced the 8th harmonic by 10 dB while the 7th and 9th harmonics were reduced by 10 dB.

This result can be extended to recording engineering and artificial intelligence music production, such as by helping manufacturers of musical instrument sound source software and electronic effector software to improve the timbre of their products, and by providing manufacturers of audio processing software containing artificial intelligence algorithms with a new timbre optimization algorithm.

## Data availability statement

The original contributions presented in this study are included in the article/supplementary material, further inquiries can be directed to the corresponding author.

## Ethics statement

The studies involving human participants were reviewed and approved by the College of Music Ethics Committee, Southwest University, China. The patients/participants provided their written informed consent to participate in this study.

## Author contributions

YC: study design and execution, data interpretation and analysis, and manuscript. YL: data statistics. GC: data analysis and statistics. XZ: study design, manuscript drafting, and reviewing. All authors contributed to the article and approved the submitted version.
